# Longevity of Brain-Workers

**Published:** 1881-12

**Authors:** 


### LONGEVITY OF BRAIN-WORKERS.

When unaccompanied by worry brain-work is essentially and inherently healthy. Brain-workers have less worry and more positive comfort and happiness than muscle-workers. Brain-workers live under better sanitary conditions than muscle-workers. The nervous temperament, which usually predominates in brain-workers, is antagonistic to fatal acute inflammatory disease and favorable to long life. Brain-workers can adapt their labor to their moods and hours. —*Beard.*

